# Platinum(II) Terpyridine Anticancer Complexes Possessing Multiple Mode of DNA Interaction and EGFR Inhibiting Activity

**DOI:** 10.3389/fchem.2020.00210

**Published:** 2020-04-28

**Authors:** Chaoyang Li, Fengmin Xu, Yao Zhao, Wei Zheng, Wenjuan Zeng, Qun Luo, Zhaoying Wang, Kui Wu, Jun Du, Fuyi Wang

**Affiliations:** ^1^Beijing National Laboratory for Molecular Sciences, CAS Key Laboratory of Analytical Chemistry for Living Biosystems, Beijing Centre for Mass Spectrometry, Institute of Chemistry, Chinese Academy of Sciences, Beijing, China; ^2^Key Laboratory of Functional Molecular Solids, The Ministry of Education, Anhui Laboratory of Molecular-Based Materials, College of Chemistry and Materials Science, Anhui Normal University, Wuhu, China; ^3^University of Chinese Academy of Sciences, Beijing, China

**Keywords:** anticancer agents, platinum terpyridine complex, EGFR inhibition, DNA binding, gefitinib, ToF-SIMS cell imaging

## Abstract

Platinum(II) terpyridine complexes has attracted increasing attention as they have displayed great potential as antitumor agents due to their high intercalation affinity with nucleic acids. Epidermal growth factor receptor (EGFR) is often overexpressed in various tumor cells, leading to uncontrolled growth of tumor, and is regarded as an important target for developing novel antitumor drugs. Herein, we report four platinum(II) terpyridine complexes bearing EGFR inhibiting 4-anilinoquinazoline derivatives as potent multi-targeting antiproliferation agents against a series of cancer cells. EGFR inhibition assay revealed that these complexes are highly potent EGFR inhibitors. But competitive DNA binding assay and docking simulations also suggested that these complexes exhibited multiple modes of DNA interaction, especially great affinity toward DNA minor groove. Finally, cellular uptake and distribution measurements by inductively coupled plasma mass spectrometry (ICP-MS) and time-of-flight secondary ion mass spectrometry (ToF-SIMS) demonstrated that both nucleus DNA and membrane proteins are important targets for their anticancer mechanisms. The complexes herein can therefore be regarded as promising multi-targeting anticancer agents.

## Introduction

Since the approval of cisplatin as an anticancer agent in the late 1970s, it has been used worldwide in clinics as an important chemotherapeutic drug for the treatment of cancer (Hanif and Hartinger, 2018; Kenny and Marmion, 2019). This breakthrough advancement further inspired the development of other platinum anticancer agents, such as two worldwide-approved drugs, carboplatin, and oxaliplatin, to overcome the downsides of cisplatin. However, the clinical application of three generations of platinum anticancer drugs still has limitations due to their intrinsic and acquired drug resistance and severe side effects such as nephrotoxicity, neurotoxicity, ototoxicity, gastrointestinal toxicity, and emetogenesis (Qi et al., 2019). Therefore, increasing attention was paid to the development of metal complexes that have mechanisms of action different from those of cisplatin. One strategy is to develop Pt^IV^ complexes as prodrugs, such as the photoactive Pt^IV^-azido anticancer complexes (Farrer et al., 2010; Shi et al., 2018, 2019; Wang et al., 2019). Another very important strategy is to change the geometry of the coordinated ligands around the Pt^II^ center, which has produced a panel of complexes with distinct activities with respect to cisplatin (Johnstone et al., 2016). In recent years, Pt^II^ terpyridine anticancer complexes (Mitra et al., 2014; Harper and Aldrich-Wright, 2015; Choroba et al., 2019) and a photoactive Pt^IV^ terpyridine anticancer complex (Canil et al., 2019) have attracted much attention for their potential as ideal anticancer candidates due to their high activity to bind to nucleic acids (Lippard, 1978; Shi et al., 2016; Chai et al., 2019) and G-quadruplex DNA (Morel et al., 2019). Besides direct coordination of metal center to DNA, non-covalent interactions such as groove binding and intercalation by the terpyridine ligands also play important roles in interactions with DNA (Keene et al., 2009), which indicates different mechanisms of action compared to cisplatin and its analogs.

The epidermal growth factor receptor tyrosine kinase (EGFR-TK) plays key roles in cell growth, proliferation, and differentiation through kinase signaling pathways by phosphorylation of the intracellular tyrosine kinase domain. It has been demonstrated to be overexpressed in many kinds of tumor cells and to be important for tumor cell proliferation, apoptosis inhibition, angiogenesis, and metastasis, which makes it an important target for the development of anticancer agents (Ciardiello and Tortora, 2001). Thereby, a series of EGFR inhibitors, in particular, 4-aminoquinazoline derivatives, have been developed and applied clinically in past decades, such as gefitinib, erlotinib, lapatinib, and vandetanib, for the treatment of non-small-cell lung cancer, colon cancer, stomach cancer, liver cancer, breast cancer, etc. (Fukuoka et al., 2003; Das and Hong, 2019). They can selectively block the signal transduction pathways of the kinase-stimulated tumor progression, thus inhibiting the growth of tumors. However, as cancer is a multigenic disease, upregulation of alternative signaling pathways or mutation of alleles may lead to acquired resistance of kinase inhibitors. Therefore, multiple-targeting anticancer agents are in urgent need.

With the advances in deeper understanding of human genome and proteome, a rational design of multiply targeted anticancer drugs has become possible by selectively disrupting multiple carcinogenic biological processes. Multi-targeting drugs can act at two or more targets so as to enhance the therapeutic effect and lower the chance for acquired resistance (Zheng et al., 2016, 2017). A number of multi-targeting anticancer drugs are currently in the clinical phase or on trials (Tao et al., 2015), such as sorafenib (Wilhelm et al., 2006) and sunitinib (Bello et al., 2006). Multi-targeting platinum and ruthenium drugs of various types have been developed in recent years (Kenny and Marmion, 2019). Besides DNA, metal complexes can also be designed to selectively target cancer cells, cell organelles such as mitochondria, and/or enzymes, peptides, and intracellular proteins. A series of ruthenium anticancer complexes were reported to have both potent enzyme inhibition and DNA interaction activities (Kurzwernhart et al., 2012; Kilpin and Dyson, 2013). A platinum-based multi-targeting anticancer complex was also demonstrated to exhibit both DNA binding and anti-inflammatory activity (Cheng et al., 2014; Pathak et al., 2014). In the previous work of our group, we developed a panel of dual-targeting metal-based anticancer complexes using the “pharmacophore conjugation” strategy by coupling a ruthenium complex moiety with a gefitinib pharmacophore (Zheng et al., 2013; Ji et al., 2014; Du et al., 2015, 2016; Zhang et al., 2015). Recently, a series of platinum(II) (Yang et al., 2018), gold(I) (Yang et al., 2015), and cobalt(III) (Karnthaler-Benbakka et al., 2014) complexes containing gefitinib derivatives have also been reported to have anticancer activity. These multi-targeting anticancer complexes displayed both DNA-interacting and EGFR-inhibiting activities. In this work, being inspired by the potent anticancer activity and unique mechanisms of action of the platinum(II) terpyridine complexes, we designed and synthesized a series of multi-targeting anticancer complexes containing both platinum(II) terpyridine moiety and EGFR-inhibiting 4-anilinoquinazoline moiety. The anticancer activity, interaction with both EGFR and DNA as potential targets, and subcellular distribution of these target complexes were studied in detail, which suggests their promising prospect as novel multi-targeting anticancer candidates.

## Materials and Methods

The synthesis and characterization of complexes **1**–**4** are in the supporting information.

### DNA Interaction

Complexes **2** and **4** were dissolved in DMSO to yield a panel of 0.25, 0.5, 1.0, 1.5, 2.0, 3.0, 4.0, and 5.0 mM solutions. The calf thymus DNA (ct-DNA) was dissolved in Tris-HCl buffer (pH = 7.4) to give a 200 mM solution. Ethidium bromide (EB) (10 mM, 2 μl) or Hoechst 33342 (4 mM, 5 μl) and ct-DNA (200 mM, 978 or 975 μl) was incubated at 310 K for 0.5 h. Then the DMSO solution of complexes (20 μl) was added to the resulting solution and kept incubated. After 2 h, each reaction mixture was measured on an F-4500 fluorescence spectrophotometer (HITACHI) with excitation wavelength at 500 nm and emission spectra from 520 to 700 nm for EB mixed solution and excitation wavelength at 370 nm and emission spectra from 400 to 650 nm for Hoechst 33342 mixed solution. A modified Stern–Volmer plot (Sarwar et al., 2015) was employed to evaluate the affinity of complexes toward DNA. The fluorescence intensities were recorded with different concentrations of complex **2** or **4**, and *K*_sv_ was fitted using Origin 8.0 (OriginLab Corporation, USA) by the following equations:

(1) $$\begin{array}{l} F_{0}/F=1+K_{\mathrm{sv}}\left[Q\right] \end{array}$$

where *F*_0_ and *F* are the fluorescence intensities of the EB–ct-DNA or Hoechst–ct-DNA complex recorded before and after adding complex **2** or **4**, respectively. [*Q*] is the final concentration of complex **2** or **4** in the reaction mixture.

### EGFR Inhibition

Enzyme-linked immunosorbent assay (ELISA) was employed to evaluate the inhibition of compounds against EGFR. The ELISA screening was performed following the instruction provided by the supplier of the assay kits (no. 7909, Cell Signaling Technology, Inc.). An aliquot (10 μl) of the enzyme solution was added to 415 μl dithiothreitol (DTT) kinase buffer, which consists of 1.25 M DTT and 4 × HTScan® tyrosine kinase buffer [240 mM HEPES (pH 7.5), 20 mM MgCl_2_, 20 mM MnCl_2_, and 12 μM Na_3_VO_4_]. Each tested Pt compound was dissolved in dimethyl sulfoxide (DMSO) to give a 4 mM solution which was then diluted by deionized water prior to use. The ATP/peptide mixture was prepared by addition of 10 μl of 2.5 mM ATP to 125 μl of 6 μM substrate peptide and then diluted with D_2_O to 250 μl. An aliquot (12.5 μl) of the solution of a tested compound was mixed with as-prepared EGFR (kinase domain only) solution [12.5 μl, in 50% glycerol, containing 50 mM HEPES (pH = 7.6), 150 mM NaCl, 0.1% Triton, and 1 mM DTT, Sigma Chemical Company] and incubated at 298 K for 5 min, followed by addition of 25 μl of ATP/substrate mixture, and then the resulting mixture was incubated at 310 K for 1 h. The phosphorylation reaction was terminated by the addition of 50 μl/well stop buffer (50 mM EDTA, pH = 8). Each well of a microtiter plate was coated with 100 μl of 10 μg/ml streptavidin (Tianjin Biotechnology Co. Ltd., China) in carbonate–bicarbonate buffer and incubated overnight at 277 K and then blocked with 1.5% bovine serum albumin (BSA, Xinjingke Biotechnology Co. Ltd., China) in PBS/T (PBS solution containing 0.05% Tween-20) at 310 K for 2 h, followed by three times of washing with PBS/T prior to use. Then, 25 μL/well of each enzymatic reaction mixture and 75 μL/well of D_2_O were added to the plate (in triplicate) for incubation at 310 K for 1 h. Following three times of washing with PBS/T, 100 μl of primary antibody (phospho-tyrosine mouse mAb, 1:1,000 in PBS/T with 1.5% BSA) was added to each well, and the plate was incubated at 310 K for another 1 h. The plate was again washed three times with PBS/T, and then 100 μl of the secondary antibody (HRP-labeled goat anti-mouse IgG, from Zhongshan Golden Bridge Biotechnology Co. Ltd., China, diluted 1:1,000 in PBS/T with 1.5% BSA) was added to each well and incubated for 1 h at 310 K, followed by three times of washing with PBS/T. Finally, 100 μl of 3,3′,5,5′-tetramethylbenzidine (TMB, Xinjingke Biotechnology Co. Ltd., China) substrate was added to each well, and the plate was incubated at 310 K for 15 min, and then the reaction was stopped by addition of 100 μl of 2 M H_2_SO_4_ to each well, and the plate was read on the ELISA plate reader (SpectraMax M5 Molecular Devices Corporation) at 450 nm to determine the OD values. The data were processed by Origin 8.0.

### Docking Analysis

The docking studies and molecular modeling were carried out using the Surflex-Dock module of the Sybyl X 1.1 program. The crystal structure of the EGFR–erlotinib complex from the Protein Data Bank (PDB) (1M17) (Jennifer et al., 2002) was used as the leading structure to build the corresponding structures of the complexes with the EGFR in this work. All the water molecules in the EGFR–erlotinib crystal were eliminated except H_2_O 10 as it plays an important role in the formation of hydrogen bonds between erlotinib and EGFR (Nowakowski et al., 2003). After the erlotinib molecule was extracted, the docking pocket was generated at the ATP binding cleft automatically. Then complexes **1**–**4** were successively docked into the pocket. The docking scores are given as –lgK_d_, which represents the dissociation constants of the EGFR–inhibitor complexes.

### Antiproliferative Activity

The cancer cell lines HeLa, A549, MCF-7, and A431 were obtained from the Center for Cell Resource of Peking Union Medical College Hospital, except that A549 cisplatin-resistant subline A549/DDP was obtained by incubation of increased concentration of cisplatin in the culture media until the IC_50_ value toward cisplatin was above three times of that of A549 cells. The cells were maintained in DMEM (Invitrogen, USA) media supplemented with 10% fetal calf serum (HyClone, USA), 1% PS at 310 K with 5% CO_2_. The IC_50_ values were determined using 3-(4,5-dimethyl-2-thiazolyl)-2,5-diphenyl-2-H-tetrazolium bromide (MTT) assay. The cells of A549, A549/DDP, HeLa, A431, and MCF-7 were placed at a density of 3,000, 3,000, 4,000, 8,000, and 4,000 cells per well in 100 μl media in 96-well plates (Beijing BioDee BioTech Co. Ltd., China), respectively, and cultured in the medium for 24 h. The stock solutions [5 mM, except for gefitinib (10 mM) and cisplatin (1 mM)] of all tested compounds were made up fresh in DMSO before dilution in media to give the required concentration, and the final DMSO concentration in media was 2%. Then cells were exposed to each tested compound at eight concentrations for 48 h. The resulting solution was removed and washed for two times using PBS; then 100 μl cell culture medium containing MTT (0.5 mg/ml) was added to the wells and incubated at 310 K for 4 h. Finally, the MTT media was removed and 100 μl DMSO added to each well to dissolve the formazan crystals. The optical density (OD) values were measured using a microplate reader (SpectraMax M5) at the wavelength of 570 nm. The inhibition rate (IR) was calculated based on the following equation: IR (%) = [(OD_control_ – OD_compound_)/(OD_control_ – OD_blank_)] × 100%. The IC_50_ value was calculated in Origin 8.0 using the logistic regression model. All reported values were averages of six independent experiments and expressed as mean ± standard deviation (SD).

### Inductively Coupled Plasma Mass Spectrometry (ICP-MS)

Complex **2** was dissolved in DMSO to give a 10 mM stock solution. A549 cell lines were selected as an example to study the cellular uptake and distribution. Cells were seeded in the corning cellular culture dish containing 7 ml media, and the media was changed to that containing 30 μM Pt compounds when the cell coverage was more than 90%, and the final DMSO concentration was 1%. After incubation at 310 K for 24 h, the media were removed and washed three times using PBS. Then 3 ml of 0.04% EDTA in PBS was added to detach the cells for 1 min. After that, the cells were collected with PBS and washed three times using ice-cold PBS. Then the cells were divided into two parts which were used to analyze the platinum content in membrane and nucleic DNAs. The TIANamp Genomic DNA Kit, RNase A, and Bestbio-Membrane Protein Extraction kit [TIANGEN Biotech (Beijing) Co., Ltd.] were used to extract the nucleus fractions and membrane protein, respectively. The DNA concentration was measured by using UV–visible spectroscopy, and the protein concentration was determined using a BCA Protein Assay Kit [TIANGEN Biotech (Beijing) Co., Ltd.]. The resulting extractions were decomposed by 50% HNO_3_, 20% HNO_3_, and deionized water. After being completely dried at 200°C, the solid extracts were redissolved in 1% HNO_3_, and the platinum was determined by ICP-MS (Agilent 7700x, USA) (Wei et al., 2013). Cellular metal levels were expressed as nanomole Pt per milligram DNA or protein. Results are presented as the mean of four determinations for each data point and expressed as mean ± SD.

### Time-of-Flight Secondary Ion Mass Spectrometry (ToF-SIMS) Imaging

For ToF-SIMS imaging, ~10^4^ A549 cells were seeded in a 1-in. Corning cellular culture dish containing a 1 × 1-cm silicon wafer with 1.5 ml media. After the cells were cultured at 310 K for 24 h, fresh media containing 30 μM platinum complex **2** or **4** and 1% DMSO were changed and incubated for a certain time. After that, the suspension was removed, and the cells were washed three times using ammonium acetate (150 mM, pH 7.4). Then the cells were lyophilized for 24 h by an LGJ-12 lyophilizer (Beijing Songyuan Huaxing Technology Develop Co., Ltd.). ToF-SIMS imaging was conducted using a ToF-SIMS V spectrometer (ION ToF GmbH, Munster, Germany). Dual-beam depth profiling strategy was used. A 10-keV argon cluster ion beam (${\text{Ar}}_{\text{n}}^{+}$) was used as a sputter beam, which was scanned on a 300 × 300-μm^2^ area across the A549 cell surface. The current of the ${\text{Ar}}_{\text{n}}^{+}$ was ~2 nA with a lead-off time of 60 μs. A 30.0-keV ${\text{Bi}}_{3}^{+}$ beam with a 200-pA DC current, 100-ns pulse width, and 5-kHz repetition rate was applied as an analysis beam, which was scanned on a 100 × 100-μm^2^ area at the center of the ${\text{Ar}}_{\text{n}}^{+}$ crater by 256 × 256 pixels. Negative spectra were recorded and calibrated by H^−^, C^−^, and ${\text{C}}_{2}^{-}$. The images for ions corresponding to ${\text{PO}}_{3}^{-}$ (*m*/*z* = 79.18) represent the fragments of phospholipids and nuclear acids. The images of Pt-containing fragment ions [PtC_*n*_N_*n*_]^−^ (*n* = 1 or 2, *m*/*z* = 221.64 or 247.49) represent the Pt complexes. The non-interlaced mode was used for all the imaging experiments. One scan consists of a 20-circle analysis phase, a 15-s sputtering phase, and a 2-s relaxation time for charge compensation. The cells had different sizes and thickness of contamination, so the first one to two scans were discarded for the removal of contamination over the surface of the cells. Then the next five to eight scans were regarded as the signal from the membrane and cytoplasm of the cells. Finally the next 8–14 scans were regarded as the nucleus of the cells. The intensity scale bar of [PO_3_]^−^ and [PtC_*n*_N_*n*_]^−^ signals was adjusted to the same for all the images, for the convenient comparison of their intensities.

## Results and Discussion

### Synthesis and Characterization

**Scheme 1:**
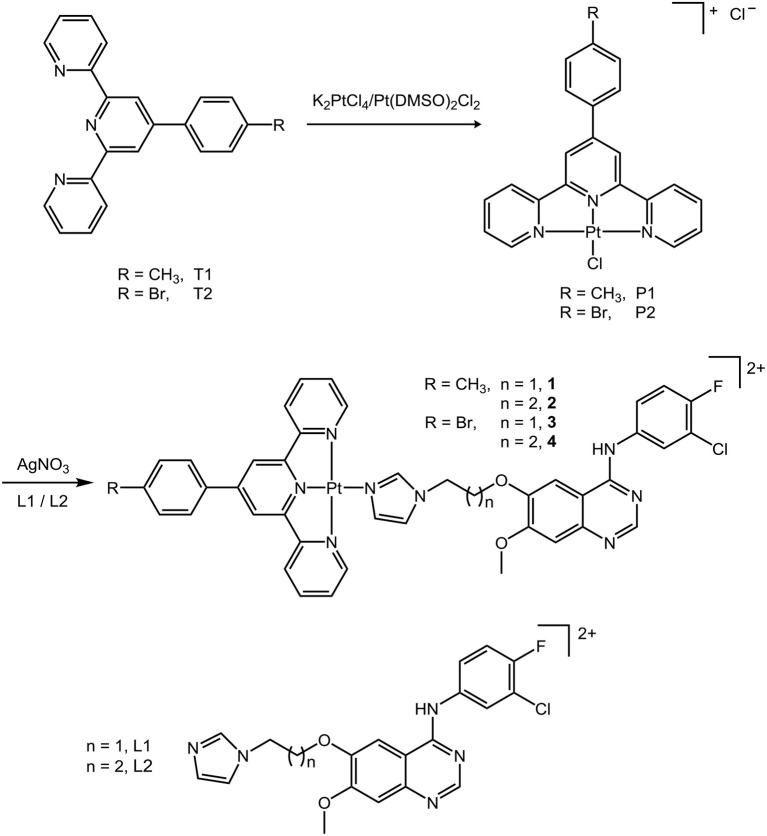
The synthesis of platinum(II) terpyridine complexes **1**–**4**.

Two derived terpyridine ligands (T1/T2) (Shi et al., 2006) and two 4-anilinoquinazoline ligands (L1/L2) were prepared following the literature methods (Du et al., 2015) with minor modification. Then T1 or T2 were reacted with Pt^II^ precursors such as K_2_PtCl_4_ or Pt(DMSO)_2_Cl_2_ to give rise to the intermediates P1 and P2 (Cummings, 2009b). After the treatment of AgNO_3_ to remove the chloride, final complexes **1**–**4** were received by the reaction of these as-prepared intermediates in methanol solution, with L1 or L2 at ambient temperature, as shown in Scheme 1. The products were characterized by HR-ESI-MS, ^1^H-NMR, ^13^C-NMR, and elemental analysis. Details are given in the supporting information. The molecule ion of complexes **1**–**4** was found in HR-ESI-MS; all ^1^H and ^13^C were found in the ^1^H-NMR and ^13^C-NMR, respectively; and elemental analysis approved the chemical formulas of the compounds.

The stability of the Pt complexes **1**–**4** in PBS was examined by HPLC. The PBS solution of complexes **1**–**4** was prepared by diluting the freshly prepared DMSO solution of the corresponding complexes in PBS, with 1% DMSO in the final solution. The HPLC chromatograms were recorded both immediately and after incubating at ambient temperature for 48 h. As shown in Figure S1, neither significant hydrolysis nor DMSO substitution was observed. This result suggested that the Pt complexes in this work do not hydrolyze or decompose in the biosystem, which also minimizes the chance of covalent bonding toward DNA bases.

### DNA Interaction

DNA was widely studied as a target of cytotoxic Pt terpyridine complexes, which selectively bind to DNA by covalent or non-covalent interactions (Lippard, 1978; Cummings, 2009a; Shi et al., 2016; Chai et al., 2019). The hydrolysis of the cleaving group of Pt^II^ terpyridine complexes can result in covalent binding with nucleobases, while the terpyridine ligands may non-covalently interact with DNA by intercalating into the base pairs. Moreover, the DNA minor groove binding for some anticancer compounds often plays an important role in antitumor activity (Avendaño and Menéndez, 2015). A series of Ru complexes with the EGFR inhibiting 4-anilinoquinazoline moiety have also been reported (Du et al., 2015, 2016). Since complexes **1**–**4** are very stable and inert to hydrolysis in aqueous solution, covalent binding to DNA may not be involved. In order to study the interaction with DNA of these complexes, therefore, we performed a competitive fluorescent titration assay to evaluate their ability of intercalation and minor groove binding with ct-DNA using EB and Hoechst 33342® (Hoechst), respectively.

**Figure 1:**
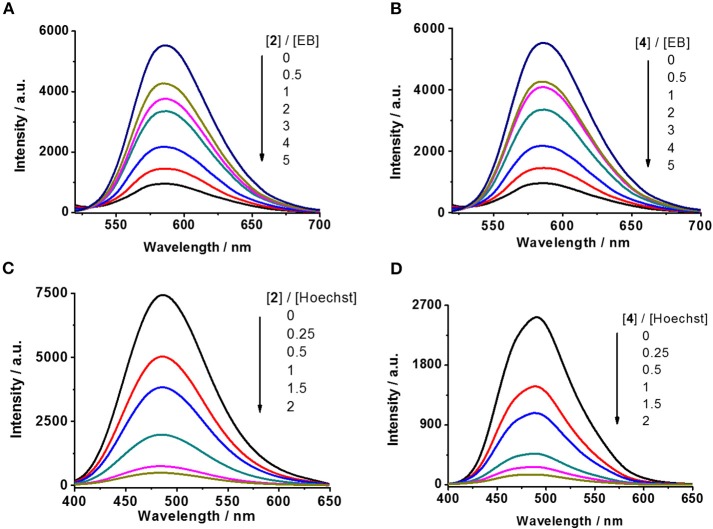
Competitive displacement assays by fluorescence titration. **(A,B)** DNA–EB complex against complexes **2** and **4**, respectively (λ_ex_ = 500 nm). **(C,D)** DNA–Hoechst complex against complexes **2** and **4**, respectively (λ_ex_ = 370 nm).

The fluorescence of EB is quenched in aqueous solution but can be restored upon intercalation with DNA base pairs (Liu and Sadler, 2011). Similarly, the fluorescence of the other probe Hoechst is retrieved when it binds to DNA at the minor groove (Guan et al., 2006). The compounds that are able to intercalate DNA or bind to the minor groove may decrease the fluorescence intensity of the DNA–EB or DNA–Hoechst complex, respectively (Ortmans et al., 2004). When complex **2** or **4** was titrated to the aqueous solution of the DNA–EB or DNA–Hoechst complex, a successive decrease of the emission intensities was observed in both cases, as shown in Figures 1A–**D**, indicating that **2** and **4** could interact with DNA through both intercalation and minor groove binding.

**Figure 2:**
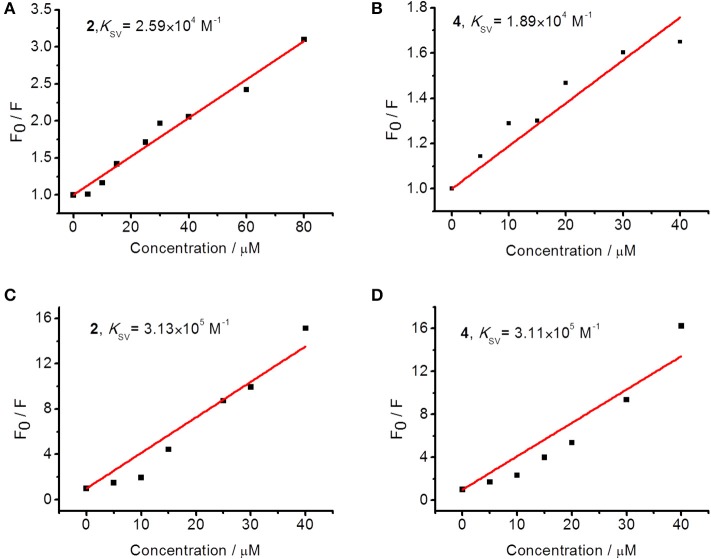
Stern–Volmer plots for the competitive displacement assays measured by the quenching of fluorescence intensity by **(A,B)** DNA–EB complex and **(C,D)** DNA–Hoechst complex by **2** or **4**, respectively.

Furthermore, quenching constant (*K*_sv_, Stern–Volmer constant, Sarwar et al., 2015) was calculated to evaluate the binding affinity of these complexes with DNA. As shown in Figure 2, the *K*_sv_ of complexes **2** and **4** are 2.59 × 10^4^ and 1.89 × 10^4^ M^−1^, respectively, in replacing EB from ct-DNA and 3.13 × 10^5^ and 3.11 × 10^5^ M^−1^, respectively, in replacing Hoechst from ct-DNA. Various substitutions of Br and methyl on the 4-benzene group of terpyridine ligands were initially designed to evaluate the structure–activity relationship for the interaction of the produced Pt complexes with DNA. This result suggests that complex **2** has similar affinity in minor groove binding and intercalation with DNA to that of complex **4**, which indicates that the substitution of Br or methyl does not substantially affect their interaction with DNA. But the affinity of complexes **2** and **4** in minor groove binding is ~10 times higher than that for intercalation with DNA, indicating that minor groove binding to DNA is the major mode of action for these complexes.

### EGFR Inhibition

The EGFR-inhibiting activity of complexes **1**–**4** was evaluated using ELISA. Gefitinib, a clinical anticancer drug and a potent EGFR inhibitor, was employed as a positive control. The results are listed in Table 1. Complexes **1**–**4** showed very high EGFR-inhibiting activity with IC_50_ values of ~10 nM, ~10 times lower than that of gefitinib (IC_50_ = 94.0 nM). Their inhibition potency toward EGFR is also much higher than that for their precursor ligands L1 (IC_50_ = 57.4 nM) and L2 (IC_50_ = 69.6 nM) (Du et al., 2016), indicating that tethering a Pt^II^ terpyridine group to an anilinoquinazoline moiety promoted the inhibition against EGFR. It is worth mentioning that the IC_50_ value of gefitinib to EGFR was reported to be 33 nM in the presence of 5 μM of ATP (Muhsin et al., 2003), simply because gefitinib is an ATP-competitive EGFR inhibitor, so a higher level of ATP can offset its activity. These results unambiguously demonstrated the high EGFR-inhibiting activity of complexes **1**–**4**. The high inhibition activity against EGFR and high minor groove binding affinity of these complexes suggest their multi-targeting activity. Specifically, complexes **2** and **4** showed slightly higher inhibitory activity against EGFR than did complexes **1** and **3**. The insignificant differences of the IC_50_ values of complexes **1**–**4** suggest that neither the variation of the length of linkers nor the substitution of methyl with bromine at the 4′ position of the terpyridine had a substantial effect on their EGFR inhibition. The Ru-arene complexes in conjugation with an anilinoquinazoline moiety reported in our group previously displayed high dependency between the length of linkers and the inhibition against EGFR (Zheng et al., 2013; Du et al., 2015; Zhang et al., 2015). The longer linker is usually correlated with higher inhibitory activity. But herein, this relationship for complexes **1**–**4** is not definite. This is possibly due to the different sizes, shapes, and conformation of the Pt-tpy groups and Ru-arene groups.

**Table 1:** IC_50_ for inhibition toward EGFR activity and the growth of human carcinoma cell lines of complexes **1**–**4**.

**Compound**	**IC_**50**_ to EGFR (nM)^**a**^**	**IC**_****50****_ **(μM) to cell lines exposed to tested compounds for 48 h**					
		**A549**	**A549/DDP**	**RF^**d**^**	**A431**	**MCF-7**	**HeLa**
**1**	9.2 ± 0.6	13.2 ± 1.8	28.9 ± 1.7	2.2	7.7 ± 1.8	20.3 ± 2.7	29.8 ± 5.0
**2**	7.0 ± 3.0	6.5 ± 2.5	13.5 ± 1.8	2.1	1.4 ± 0.4	27.7 ± 2.2	19.4 ± 5.6
**3**	10.9 ± 3.0	12.2 ± 2.2	32.0 ± 1.6	2.6	7.4 ± 0.4	24.8 ± 1.1	13.4 ± 1.3
**4**	9.6 ± 2.5	11.6 ± 1.1	26.6 ± 3.0	2.3	7.9 ± 3.2	19.9 ± 2.7	39.4 ± 0.1
Gefitinib	94.0 ± 3.1^b^	16.0 ± 1.0	24.9 ± 1.2	1.6	12.6 ± 1.3	19.9 ± 2.0	30.8 ± 3.0
Cisplatin	NT^c^	10.1 ± 1.6	30.9 ± 1.1	3.1	11.1 ± 0.4	15.6 ± 0.8	12.9 ± 0.6

Gefitinib and cisplatin were used as positive controls.

a The IC_50_ data against EGFR were determined by ELISA in the presence of 200 μM of ATP.

b These data are adapted from Du et al. (2016).

c Not tested.

d *RF (resistance factor) = IC_50_ (A549/DDP)/IC_50_ (A549)*.

### *In silico* Docking Analysis

**Figure 3:**
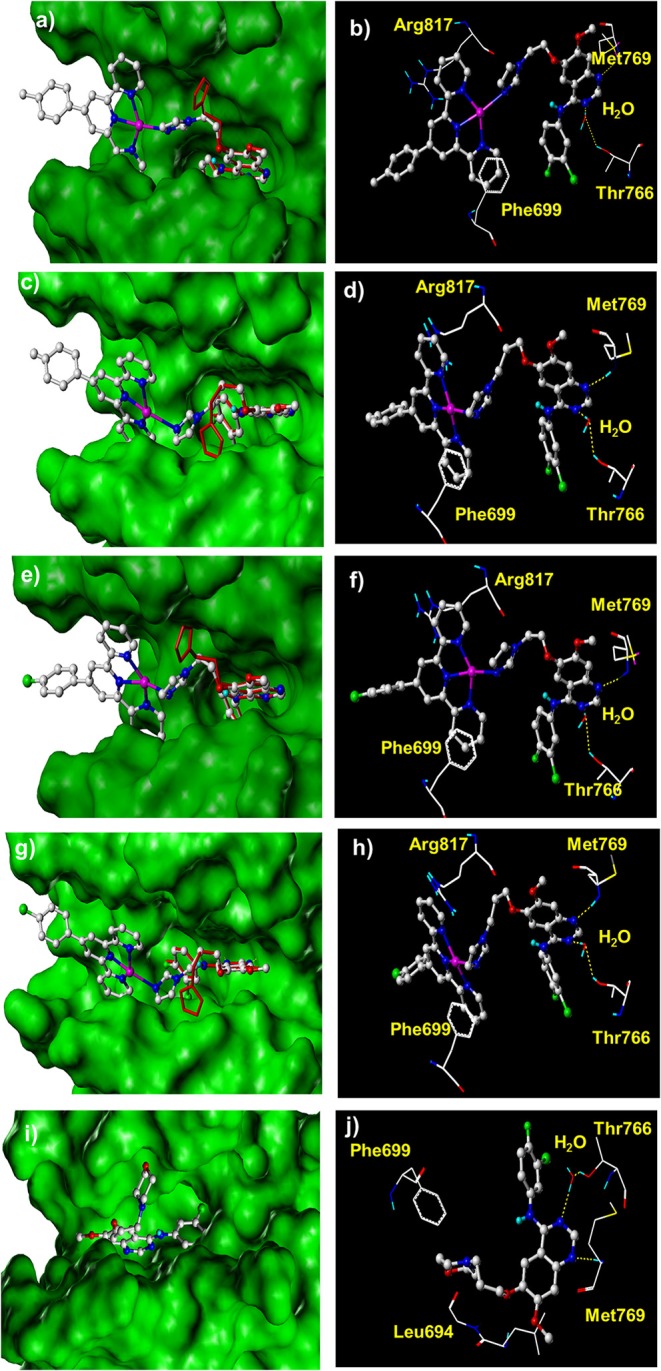
The docked conformation of compounds **1**–**4** with comparison with their corresponding ligands and gefitinib at the ATP binding cleft of EGFR kinase. **(a,b)** Complex **1**; **(c,d)** complex **2**; **(e,f)** complex **3**; **(g,h)** complex **4**; **(i,j)** gefitinib. The red molecules in stick model in **(a,c,e,g)** are the corresponding ligands L1 and L2.

For a better understanding of the mechanisms of action of these synthesized complexes with their potential targets EGFR and DNA, an *in silico* molecular docking simulation assay was performed using Surflex-Dock, an automatic docking program available in Sybyl-X 1.1 (Tripos Inc.) that uses complementary structural and topological methods to evaluate the binding affinity between the receptor and ligand. The crystal structures of EGFR were received from the PDB under the code 1M17 (Jennifer et al., 2002). After the optimization of the structures, including extracting the existing binding ligand, adding the hydrogen atoms, and removing the unnecessary water molecules, complexes **1**–**4** were docked into the binding pockets generated at the ATP binding cleft of EGFR. The binding affinity is given as docking scores (expressed as –lg*K*_d_) as shown in Table 2. The binding conformations for complexes **1**–**4** and gefitinib with EGFR are shown in Figure 3. Their corresponding ligands L1 and L2 were also merged for comparison. These complexes exhibited high affinity to the ATP binding pocket of EGFR. The docking scores are between 8.82 and 8.02, which are much higher than those for gefitinib (6.80), L1 (6.43), and L2 (6.62) (Zhang et al., 2015; Du et al., 2016). These results are consistent with the data from the EGFR inhibition assay showing that complexes **1**–**4** inhibit EGFR 10 times better than they did for gefitinib, L1, and L2 (Table 1). All the conformations of these dockings maintained two key hydrogen bonds: one between the N1 of the quinazoline ring and Met769 of EGFR and another between N3 of quinazoline and Thr766 using a water molecule as a bridge. These two hydrogen bonds were regarded as key interactions between EGFR and its inhibitors (Jennifer et al., 2002). Notably, complexes **1**–**4** have an extra π-π interaction between a pyridine group of the Pt moieties and Phe699 of EGFR, as shown in Figures 3a–h. By comparison, gefitinib, L1, and L2 could not form such an interaction (Figures 3i,j). This is possibly the main reason for the higher affinity of complexes **1**–**4** toward EGFR than that toward gefitinib, L1, and L2. In addition, complexes **2** and **4** are more favorable to insert to the ATP binding pocket than are complexes **1** and **3**, respectively. This phenomenon was to a certain degree consistent with but more definite than the aforementioned ELISA results that verified the rationale of the docking process. This result suggested that a longer flexible linker (2C−3C) between the Pt-containing moiety and the EGFR-inhibiting moiety can lower the steric hindrance, thus leading to greater affinity toward EGFR.

**Table 2:** The docking scores of compounds **1**–**4** and gefitinib on EGFR and the double-strand model DNA.

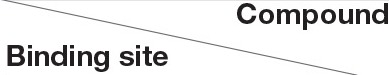	**1**	**2**	**3**	**4**	**L1**	**L2**	**Gefitinib**
EGFR ATP binding pocket	8.02	8.61	8.07	8.82	6.43	6.62	6.80 (Zhang et al., 2015)
DNA minor groove	7.06	7.33	7.48	6.57	–	–	4.05
DNA intercalation	5.55	5.70	5.65	5.36	–	–	–

**Figure 4:**
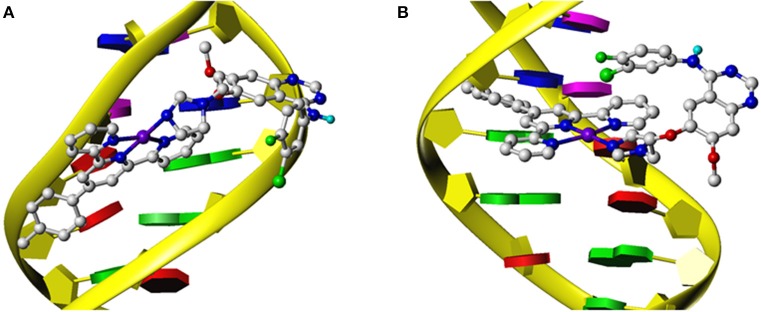
The docking models for complex **1** and a double-strand DNA constructed by the Surflex-Dock module of Sybyl X 1.1 that illustrate the interaction at minor groove **(A)** or by intercalation **(B)**.

The docking assay toward DNA at either the minor groove or the designated gap of the base pairs was also carried out. The crystal structures of a double-strand model DNA [I: 5′-(CGCGAATTCGCG)-3′, complementary II: 5′-(GCGCTTAAGCGC)-3′)] were retrieved from PDB under the code 1BNA. Complexes **1**–**4** were docked into the minor groove or the designated gap of the base pairs of DNA, respectively. The docking scores shown in Table 2 suggested that complexes **1**–**4** exhibited strong affinity with the minor groove of the DNA. The docking scores ranged from 6.57 to 7.48, much higher than that for gefitinib (4.05). Figure 4A depicts a typical binding conformation of complex **1** to the minor groove of DNA through hydrophobic interaction and hydrogen bonds. Figure 4B describes the typical intercalation confirmation between complex **1** and the double-strand DNA. The DNA intercalation docking scores of complexes **1**–**4** (Table 2) suggested that the DNA intercalation affinities were much weaker than those for minor groove binding. These data suggested that minor groove binding is a primary mode when these complexes interact with DNA, being consistent with the results of fluorescent titration competition assay results described above.

### Antiproliferation Activity

**Figure 5:**
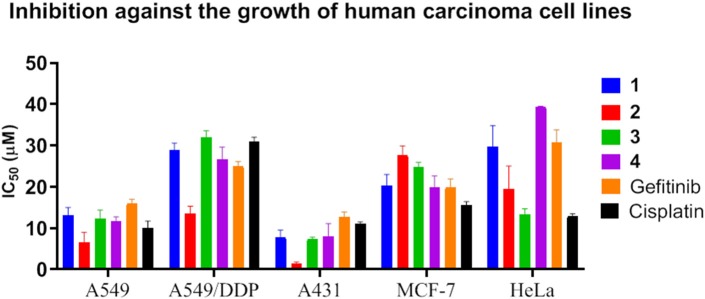
IC_50_ for inhibition against the growth of human carcinoma cell lines by complexes **1**–**4**. Data for gefitinib and cisplatin were also plotted as controls.

MTT assay was employed to evaluate the antiproliferation activity of the platinum complexes **1**–**4** toward the various cell lines, including human squamous cell carcinoma (A431), human cervical cancer (HeLa), human breast cancer (MCF-7), human non-small-cell lung carcinoma (A549), and A549 cisplatin-resistant subline (A549/DDP). Two widely applied clinical anticancer drugs gefitinib and cisplatin were also used for comparison, as gefitinib is a rationally designed EGFR inhibitor and cisplatin is a traditional cytotoxic anticancer drug. The results are shown in Table 1 and Figure 5. Complexes **1**–**4** all displayed potent antiproliferation activity toward the cancer cell lines with IC_50_ values below 50 μM. For A549 and A549/DDP cells, complexes **1**–**4** have similar anticancer activity compared to cisplatin and gefitinib, while complex **2** is the most potent. The resistance factors (RF) are ~3 for cisplatin, but for complexes **1**–**4**, the RFs are lower than 3. This to a certain extent supports that the multi-targeted complexes could overcome the cross-resistance with cisplatin.

For A431 cells that express the highest level of EGFR, complexes **1**–**4** showed very potent antiproliferation activity, with IC_50_ values mostly below 10 μM and the minimal one down to ~1 μM (complex **2**), much lower than those of cisplatin and gefitinib, which indicates that EGFR inhibition and DNA interaction are both taking effect. For MCF-7 and HeLa cells, these complexes also demonstrated antiproliferation activity comparable to that of gefitinib and cisplatin. It is notable that A431 cells expressed the highest level of EGFR and MCF-7 cells the lowest among these cell lines. Accordingly, complexes **1**–**4** displayed higher anticancer activity against A431 cells than did gefitinib and cisplatin. These results suggest that both EGFR inhibition and DNA interaction are involved in the mechanisms of action of the anticancer activity of complexes **1**–**4**. This is consistent with the molecular simulation data showing that complexes **1**–**4** have high affinity to both EGFR and DNA minor groove.

The antiproliferation activity demonstrated that complexes **1**–**4** are potent anticancer agents, especially against lung cancer. The difference of the anticancer activity of the Pt complexes between the Br- and CH_3_-substituted terpyridine ligands was trivial, which is possibly due to their weaker affinity for intercalation than for minor groove binding, as demonstrated in Figures 1, 2. Moreover, multiple mechanisms could benefit from circumventing the cross-resistance toward the traditional anticancer drugs.

### Cellular Uptake and Distribution

The cellular uptake and subcellular distribution of the complexes in this work were studied by ICP-MS and ToF-SIMS. A549 cells were incubated with complex **2** (30 μM) for 24 h, and the level of platinum binding to cell membrane protein and DNA was determined by ICP-MS. The results indicated that 0.43 ± 0.07 nmol Pt incorporated to 1 mg membrane protein and 0.19 ± 0.03 nmol Pt to 1 mg DNA, indicating this complex can not only distribute at the cell membrane, possibly interacting with EGFR, but also pass through the membrane and interact with the DNA in the cell nucleus. This result again demonstrates the dual-targeting property of the complex.

ToF-SIMS imaging was employed to further study the intracellular distributions of these complexes. ToF-SIMS is a powerful micro-analysis tool for the surface of materials, which is recently applied in the mass spectrometry imaging of biological samples, such as single cell and tissue (Liu et al., 2017; Oomen et al., 2019; Ranjbari et al., 2019). Complexes **2** and **4** were used as examples, which were incubated with A549 cells for 3, 12, or 24 h, before ToF-SIMS imaging was carried out. An analysis beam and a sputter beam were used in an non-interlaced mode following a revised method reported previously by our group (Liu et al., 2017). As the cells were in different sizes and had different thicknesses of contamination, ToF-SIMS imaging data were extracted in three steps. In the first step, one or two sputter scans were regarded as the cleanness of the surface contamination. Then five to eight circles of sputtering and imaging scans were alternatively performed. The mass spectra and the images obtained during this step can be regarded from the surface of the cell and represent the chemical composition of the cell membrane and cytoplasm. In the third step, 8–14 circles of sputtering and imaging were applied to collect the mass spectra from deep inside of the cell; the image of this step can be regarded to represent the components of the nucleus. The number of scans in each step varies, which depends on the thickness and size of the individual cell. A total scan of 25–30 were usually needed to analyze a single cell until the cells were sputtered out, and a detailed description can be found in the experimental section.

**Figure 6:**
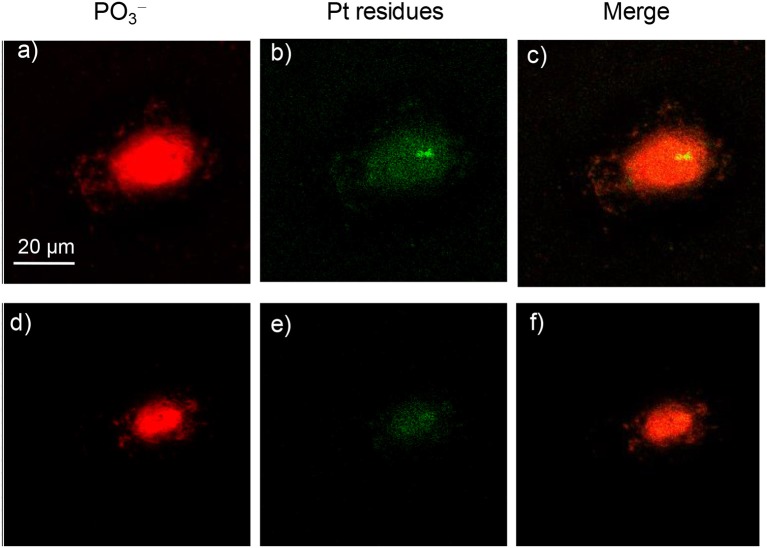
ToF-SIMS images of an A549 cell exposed to 30 μM platinum complex **2** at 310 K for 3 h. **(a,d)** Images for [PO_3_]^−^, which correspond to the fragment ions of phospholipids and nucleic acids. **(b,e)** Images for Pt-containing fragment ions [PtC_*n*_N_*n*_]^−^ (*n* = 1 or 2) arising from complex **2**. **(c,f)** The corresponding overlapped images of the above. **(a–c)** correspond to the accumulation of signal from scans 2–7 (cell membrane and cytoplasm), and **(d**–**f)** correspond to scans 8–15 (cell nucleus).

**Figure 7:**
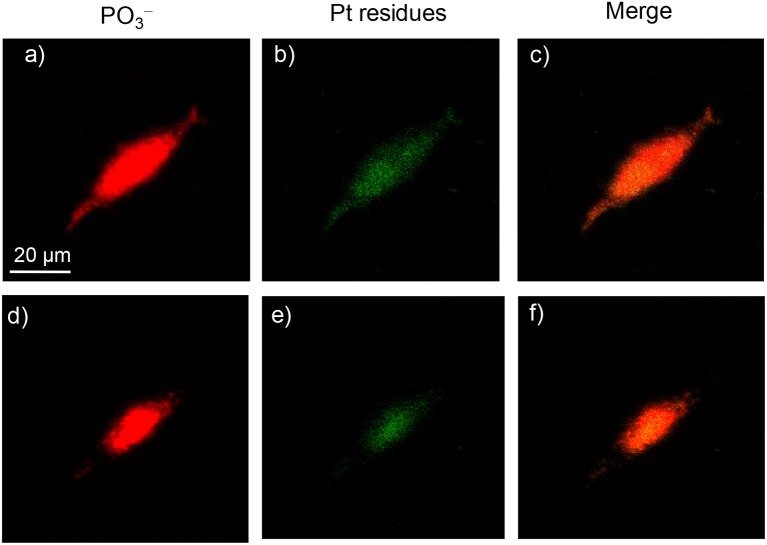
ToF-SIMS images of an A549 cell exposed to 30 μM platinum complex **2** at 310 K for 24 h. **(a,d)** Images for [PO_3_]^−^, which correspond to the fragment ions of phospholipids and nucleic acids. **(b,e)** Images for Pt-containing fragment ions [PtC_*n*_N_*n*_]^−^ arising from complex **2**. **(c,f)** The corresponding overlapped images of the above. **(a–c)** correspond to the accumulation of signal from scans 3–10 (cell membrane and cytoplasm), and **(d–f)** correspond to scans 11–24 (cell nucleus).

In ToF-SIMS imaging, the [PO_3_]^−^ anion (*m*/*z* = 79.18) could be produced from the fragmentation of phospholipids and nucleic acids. The images of [PO_3_]^−^ profile the cell membrane in the images of the surface and nucleus in the images of deep inside the cell. In comparison, the characteristic platinum-containing fragment ions, [PtCN]^−^ and [PtC_2_N_2_]^−^, represent the distribution of the platinum complexes in the cells. The intensity scale bars of [PO_3_]^−^ and [PtC_n_N_n_]^−^ signals were adjusted to the same for all the images, for the convenient comparison of their intensities. As shown in Figure 6, when A549 cells were incubated with complex **2** for only 3 h, signals from platinum-containing fragments were observed more in the cell membrane/cytoplasm and less in the nucleus (Figures 6b,e). This demonstrated that complex **2** was mostly accumulated at the cell membrane/cytoplasm and possibly interact with the membrane proteins such as EGFR. When complex **2** was incubated with A549 cells for 24 h, as shown in Figure 7, more Pt complexes could be found both in the nucleus and in the membrane/cytoplasm, which suggested that after a long incubation, complex **2** could penetrate the membrane and enter the nucleus, possibly interacting with the DNA.

**Figure 8:**
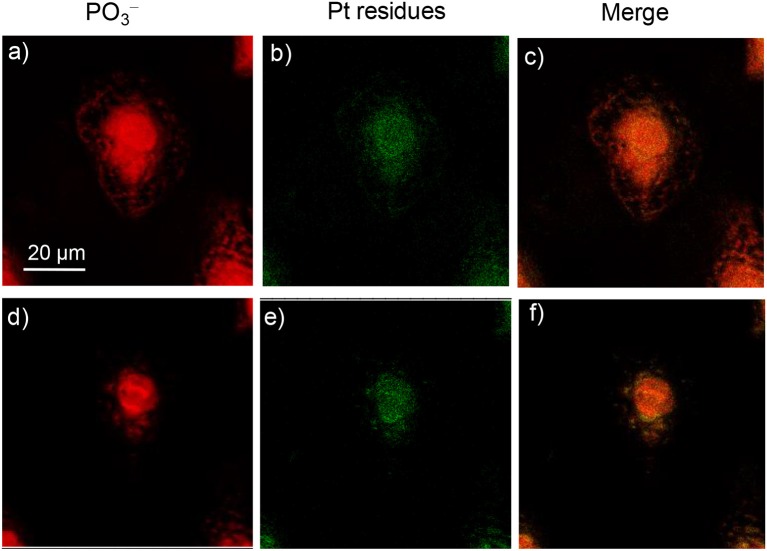
| ToF-SIMS images of an A549 cell exposed to 30 μM platinum complex **4** at 310 K for 12 h. **(a,d)** Images for [PO_3_]^−^, which correspond to the fragment ions of phospholipids and nucleic acids. **(b,e)** Images for Pt-containing fragment ions [PtC_*n*_N_*n*_]^−^ arising from complex **4**. **(c,f)** The corresponding overlapped images of the above. **(a**–**c)** correspond to the accumulation of signal from scans 2–6 (cell membrane and cytoplasm), and **(d–f)** correspond to scans 7–20 (cell nucleus).

**Figure 9:**
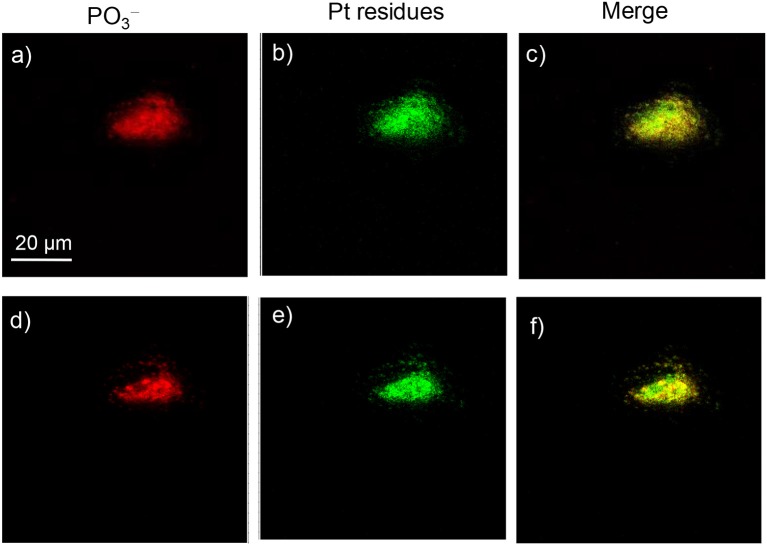
ToF-SIMS images of an A549 cell exposed to 30 μM platinum complex **4** at 310 K for 24 h. **(a,d)** Images for [PO_3_]^−^, which correspond to the fragment ions of phospholipids and nucleic acids. **(b,e)** Images for Pt-containing fragment ions [PtC_*n*_N_*n*_]^−^ arising from complex **4**. **(c,f)** The corresponding overlapped images of the above. **(a–c)** correspond to the accumulation of signal from scans 2–8 (cell membrane and cytoplasm), and **(d–f)** correspond to scans 9–18 (cell nucleus).

When complex **4** was incubated with A549 cells for 3–12 h, a very weak signal could be found in both the surface and deep inside of the cell; the images for 12 h are shown in Figure 8. Only after 24 h was a strong signal for Pt residues found in both the membrane/cytoplasm and nucleus of the cell (Figure 9). This suggested that complex **4** entered in the cell very slowly, so 24 h was needed for the accumulation of these complexes in both the membrane/cytoplasm and nucleus. These results are consistent with the above results that these anticancer complexes could interact with both DNA and membrane receptor protein and again verified the EGFR/DNA duel-targeting activity of the complexes examined in this work.

## Conclusion

In this work, four platinum(II) terpyridine complexes tethering the EGFR-inhibiting 4-anilinoquinazoline group were synthesized and characterized. These complexes exhibited 10-fold higher EGFR-inhibiting activity than the clinically used EGFR inhibitor and anticancer drug gefitinib and potent antiproliferation activity against a panel of tumor cell lines, especially lung cancer. These complexes displayed lower cross-resistance toward A549/DDP cells than that for cisplatin. For high-EGFR-expressing cells A431, complex **2** was even much more active than the clinical anticancer drugs cisplatin and gefitinib. The fluorescent titration competitive DNA binding assay and docking simulations revealed that DNA minor groove is also a very important target for these complexes. Cellular uptake and distribution measurements by ICP-MS and ToF-SIMS demonstrated that both DNA and membrane proteins are important targets of these synthesized complexes. Multiple mechanisms of the multi-targeted complexes **1**–**4** benefited from circumventing the cross-resistance with cisplatin. These data suggest a new horizon for platinum(II) terpyridine complex as potential multi-targeted antitumor agents.

## Data Availability Statement

All datasets generated for this study are included in the article/Supplementary Material.

## Author Contributions

YZ, WZh, JD, and FW designed the project. CL, FX, WZh, WZe, QL, ZW, and KW performed the experiments. YZ, WZh, and FW analyzed the data. YZ, CL, and FW wrote the paper.

## Conflict of Interest

The authors declare that the research was conducted in the absence of any commercial or financial relationships that could be construed as a potential conflict of interest.
