# Supplementary material for: Platinum(II) Terpyridine Anticancer Complexes Possessing Multiple Mode of DNA Interaction and EGFR Inhibiting Activity
Source: Front Chem. 2020 Apr 28;8:210. doi: 10.3389/fchem.2020.00210 (PMC7199514; doi:10.3389/fchem.2020.00210)
Supplement: Supplementary file 1 [file Data_Sheet_1.docx]

Supporting information for

Platinum(II) Terpyridine Anticancer Complexes Possessing Multiple Mode of DNA Interaction and EGFR Inhibiting Activity

Chaoyang Li,^a,b^ Fengmin Xu,^a,b^ Yao Zhao,*^a^ Wei Zheng,^a^ Wenjuan Zeng,^a, c^ Qun Luo,^a, c^ Zhaoying Wang,^a, c^ Kui Wu,^a^ Jun Du,*^b^ Fuyi Wang*^a, c^

^a^ Beijing National Laboratory for Molecular Sciences; CAS Key Laboratory of Analytical Chemistry for Living Biosystems; Beijing Centre for Mass Spectrometry; Institute of Chemistry, Chinese Academy of Sciences, Beijing 100190, P. R. China.

^b^ College of Chemistry and Materials Science, Key Laboratory of Functional Molecular Solids, the Ministry of Education, Anhui Laboratory of Molecular-Based Materials, Anhui Normal University, Wuhu 241000, P. R. China.

E-mail: [yaozhao@iccas.ac.cn](mailto:yaozhao@iccas.ac.cn); [fuyi.wang@iccas.ac.cn](mailto:fuyi.wang@iccas.ac.cn); [dujun@mail.ahnu.edu.cn](mailto:dujun@mail.ahnu.edu.cn)

^c^ University of Chinese Academy of Sciences, Beijing, 100049, P. R. China

**Table of Contents**

**Figure S1.**

**Supplemental Experimental Section**

**Materials and instruments**

**Synthesis and characterization**

**Figure S1.** RP-HPLC Chromatograms with detection wavelength at 254 nm of complexes a) **1**, b) **2**, c) **3** and d) **4** (100 μM) injected immediately after the DMSO solution was diluted in PBS (with 1% DMSO) (black line) and incubated at ambient temperature for 48 hours (red line).

**Supplemental Experimental Section**

**Materials and instruments**

The ligands L1, L2 were prepared as reported methods without modification.([Du et al., 2016](#_ENREF_1); [Ji et al., 2014](#_ENREF_2)) 7-methoxy-4-oxo-3,4-dihydroquinazolin-6-yl-acetate (AR grade) was purchased from Shanghai FWD Chemicals Co. (China); 1,2-dibromoethane,1,3-dibromopropane, 2-acetylpyridine, *p*-tolualdehyde and 4-bromobenzaldehyde from Beijing Ouhe Technology Co. (China). Thin layer chromatography silica gel was purchased from Yantai Institute of Chemical Industry Research (China), and silica gel column from Qingdao Jiyida Silica Reagent Manufacture (China). Organic solvents including methanol, acetonitrile, dichloromethane, diethyl ether and DMF were analytical grade and used directly without further purification. The deionized water was prepared by Milli-Q system (Mi llipore, Milford, MA).

Elemental analysis was performed on a Flash EA 1112 element analysis instrument (Thermo Quest). NMR spectra were obtained on Bruker Avance III HD 400 or a Bruker Avance 600 spectrometer (Germany). High-resolution electrospray ionization mass spectroscopy (ESI-MS) was carried out on a Q-TOF (Waters) mass spectrometer equipped with a Acquity (Waters) UHPLC system. The LC-MS data was processed on MassLynx (ver. 4.1, Waters).

**Synthesis and characterization**

**4'-*p*-tolyl-2,2':6',2''-terpyridine (T1).** 2-acetylpyridine (3.27 g, 27 mmol) and p-tolualdehyde (3.24 g, 27 mmol) were added to an aqueous solution of NaOH (2%, 50 mL) and stirred at ambient temperature for 8 h. Then 2-acetylpyridine (3.27 g, 27 mmol) and NaOH (9 g, 225 mmol) were added and the mixture was refluxed at 353 K for 8 h. The mixture was stored at 253K for 1 h and the solvent was removed by filtration. EtOH (200 mL) and NH_4_OAc (18 g, 233 mmol) were added to the residue and refluxed for 4 h. The white solid was removed by filtration immediately and the dark red filtrate was cooled to 277K to afford a yellow crystal. The crystal was recrystallized from EtOH to afford 1.98 g white crystalline needles (11.5%). Elem. anal. calcd. (%) for C_22_H_17_N_3_: C, 81.71; H, 5.30; N, 12.99; found: C, 81.78; H, 5.30; N, 13.30. ^1^H NMR (CDCl_3_, 400 MHz) *δ* (ppm): 8.74 – 8.73 (m, 4H); 8.67 (d, *J* = 8 Hz, 2H); 7.90 – 7.86 (m, *J*_1_ *= J*_2_ = 8 Hz, *J*_3_ = 1.6 Hz, 2H); 7.83 (d, *J* = 8 Hz, 2H); 7.37 – 7.31 (m, 4H); 2.43 (s, 3H). ^13^C NMR (CDCl_3_, 100.6 MHz) *δ* (ppm):156.3, 155.7, 150.3, 149.0, 139.1, 137.0, 135.5, 129.7, 127.2, 123.8, 121.5, 118.8, 29.7, 21.3. ESI-MS (*m/z*): found 324.162, ([M+H]^+^, C_22_H_18_N_3_ requires 324.150).

**4'-(4-bromophenyl)-2,2':6',2''-terpyridine (T2)**. 2-acetylpyridine (3.27 g, 27 mmol) and 4-bromobenzaldehyde (4.99 g, 27 mmol) were added to a solution of NaOH (2%, 50 mL) and stirred at ambient temperature for 8 h. Then 2-acetylpyridine (3.27 g, 27 mmol) and NaOH (9 g, 225 mmol) were added and the mixture refluxed at 353 K for 8 h. The solvent was poured and EtOH (150 mL), NH_4_OAc (18 g, 233 mmol) were added to the residue, the resulting mixture was refluxed for 4 h. The white solid was removed by filtration immediately and the filtrate was cooled to 277 K to afford a yellow solid. The solid was recrystallized from EtOH to afford 1.60 g yellow crystalline (15.5%). Elem. anal. calcd. (%) for C_21_H_14_BrN_3_·H_2_O: C, 62.08; H, 3.97; N, 10.34; found: C, 62.46; H, 3.93; N, 10.38. ^1^H NMR (DMSO-*d*_6_, 400 MHz) *δ* (ppm): 8.77 (d, *J* = 4 Hz, 2H); 8.70 – 8.67 (m, 4H); 8.07 – 8.03 (m, *J*_1_ *= J*_2_ = 8 Hz, *J*_3_ = 1.6 Hz, 2H); 7.90 (d, *J* = 8 Hz, 2H); 7.78 (d, *J* = 8 Hz, 2H); 7.54 (dd, *J*_1_ *=* 1.6 Hz, *J*_2_ = 4 Hz, 2H). ^13^C NMR (DMSO-*d*_6_, 100.6 MHz) *δ* (ppm):155.8, 154.8, 149.3, 148.3, 137.5, 136.7, 132.3, 129.0,124.6, 121.0, 117.8. ESI-MS (*m/z*): found 388.168, ([M+H]^+^, C_21_H_15_BrN_3_ requires 388.045).

**[Pt(T1)Cl]Cl (P1).** T1 (206 mg, 0.637 mmol) and K_2_PtCl_4_ (264 mg, 0.637 mmol) were added to a solution of water/acetonitrile (5 ml/15 ml), and the mixture was heated at reflux for 24 h until orange precipitate was formed. Filter the resulting mixture to produce complex P1 as an orange solid and washed with several portions of cold CH_3_CN, H_2_O and EtOH (270 mg, 72%). Elem. anal. calcd. (%) for C_22_H_17_Cl_2_N_3_Pt·H_2_O: C, 43.50; H, 3.15; N, 6.92; found: C, 43.24; H, 3.23; N, 6.86. ^1^H NMR (DMSO-*d*_6_, 400 MHz) *δ* (ppm): 8.93 (s, 2H); 8.83 (t, *J* = 8 Hz, 4H); 8.49 (t, *J* = 8 Hz, 2H); 8.12 (d, *J* = 8 Hz, 2H); 7.90 (t, *J* = 8 Hz, 2H); 7.48 (d, *J* = 12 Hz, 2H); 2.45 (s, 3H). ESI-MS (*m/z*): found 553.108, ([M – Cl]^+^, C_22_H_17_ClN_3_Pt requires 553.076).

**[Pt(T2)Cl]Cl (P2).** This complex was prepared as [Pt(T1)Cl]Cl above, by refluxing compound T2 (214 mg, 0.551 mmol) and Pt(DMSO)_2_Cl_2_ (190 mg, 0.551 mmol) in water/acetonitrile (5 ml/15 ml) for 24 h (263 mg, 73%). Elem. anal. calcd. (%) for C_21_H_14_BrCl_2_N_3_Pt·4H_2_O: C, 34.73; H, 3.05; N, 5.79; found: C, 34.82; H, 2.58; N, 5.66. ^1^H NMR (DMSO-*d*_6_, 400 MHz) *δ* (ppm): 9.03 (s, 1H); 8.92 (d, *J* = 4 Hz, 2H); 8.88 (d, *J* = 8 Hz, 2H); 8.54 (t, *J* = 8 Hz, 2H); 8.19 (d, *J* = 8 Hz, 2H); 7.96 (d, *J* = 8 Hz, 2H); 7.91 (d, *J* = 8 Hz, 2H). ESI-MS (*m/z*): found 617.032, ([M – Cl]^+^, C_21_H_14_ClBrN_3_Pt requires 616.971).

A general method for the preparation of target complexes **1** – **4** as described below in brief. AgNO_3_ (0.30 mmol) and P1/P2 (0.15 mmol) were added to 40 mL methanol, after stirring for 1 h, the precipitate of AgCl was filtered to give a clean solution, then a ligand (0.15 mmol) was added, and kept stirring overnight. The product precipitated from MeOH with an excess of NH_4_PF_6_ and the solvent was removed by filtration to afford target complexes as yellow power.

Complex **1**. 32.03 mg, 17%. Elem. anal. calcd. (%) for C_42_H_34_ClF_13_N_8_O_2_P_2_Pt·3H_2_O: C, 39.52; H, 3.16; N, 8.78; found: C, 39.55; H, 2.91; N, 8.68. ^1^H NMR (CD_3_CN, 400 MHz) *δ*(ppm): 8.60 (s, 1H); 8.51 (s, 2H); 8.46 (s, 1H); 8.43 (d, *J* = 8.0 Hz, 2H); 8.36 (t, *J* = 8.0 Hz, 2H); 8.11 – 8.09 (m, 2H); 7.93 (d, *J* = 8.0 Hz, 2H); 7.76 (d, *J* = 4.0 Hz, 2H); 7.68 – 7.65 (m, 2H); 7.53 – 7.50 (m, 4H); 7.45 (s, 1H); 7.41 (s, 1H); 7.18 (t, *J* = 8.0 Hz, 1H); 7.13 (s, 1H); 4.71 (t, *J* = 4.0 Hz, 2H); 4.58 (t, *J* = 4.0 Hz, 2H); 3.77 (s, 3H); 2.48 (s, 3H). ^13^C NMR (DMSO-*d*_6_, 150.9 MHz) *δ* (ppm): 157.93, 155.79, 154.61, 154.51, 153.92, 153.70, 152.90, 152.09, 150.99, 147.50, 147.24, 142.95, 142.33, 141.12, 136.76, 131.63, 130.10, 129.13, 128.25, 127.95, 126.32, 123.68, 122.48, 121.43, 121.39, 120.83, 118.88, 118.75, 116.59, 116.45, 108.59, 107.89, 104.38, 68.11, 56.09, 48.12, 21.03.ESI-MS (*m/z*): found 466.114, (M^2+^, C_42_H_34_ClFN_8_O_2_Pt requires 466.106); 1077.186, ([M+PF_6_]^+^, C_42_H_34_ClF_7_N_8_O_2_PPt requires 1077.177).

Complex **2**. 46.36 mg, 25%. Elem. anal. calcd. (%) for C_43_H_36_ClF_13_N_8_O_2_P_2_Pt·3H_2_O: C, 40.03; H, 3.28; N, 8.68; found: C, 39.77; H, 3.16; N, 8.62. ^1^H NMR (DMSO-*d*_6_, 400 MHz) *δ*(ppm): 9.44 (s, 1H); 9.02 (s, 2H); 8.96 (s, 1H); 8.89 (s, 2H); 8.50 – 8.47 (m, 3H); 8.16 (d, *J* = 4.0 Hz, 2H); 8.07 (s, 1H); 8.00 (s, 1H); 7.89 (m, 3H); 7.77 – 7.69 (m, 4H); 7.53 (d, *J* = 8.0 Hz, 2H); 7.33 (t, *J* = 8.0 Hz, 1H); 7.17 (s, 1H); 4.53 (s, 2H); 4.24 (s, 1H); 3.92 (s, 3H); 2.51 (s, 2H ); 2.47 (s, 3H). ^13^C NMR (DMSO-*d*_6_, 150.9 MHz) *δ*(ppm): 162.26, 157.90, 155.80, 154.80, 154.66, 153.91, 153.76, 152.79, 152.14, 151.23, 147.92, 147.23, 142.87, 142.30, 140.52, 136.70, 131.64, 130.08, 129.12, 128.50, 127.93, 126.23, 123.10, 122.71, 121.56, 120.88, 118.76, 116.60, 116.45, 108.60, 107.73, 104.54, 66.80, 55.98, 45.61, 35.74, 30.74, 28.90, 20.99 ESI-MS (*m/z*): found 473.121, (M^2+^, C_43_H_36_ClFN_8_O_2_Pt requires 473.114); 1091.206, ([M+PF_6_]^+^, C_43_H_36_ClF_7_N_8_O_2_PPt requires 1091.193).

Complex **3**. 89.12mg , 46%. Elem. anal. calcd. (%) for C_41_H_31_BrClF_13_N_8_O_2_P_2_Pt·5H_2_O: C, 35.76; H, 3.00; N, 8.14; found: C, 35.75; H, 2.87; N, 8.35. ^1^H NMR (CD_3_CN, 400 MHz) *δ*(ppm): 8.61 (s, 1H); 8.54 (s, 2H); 8.50 (s, 1H); 8.46 (d, *J* = 8.0 Hz, 2H); 8.40 (t, *J* = 8.0 Hz, 2H); 8.11 (d, *J* = 4.0 Hz, 2H); 7.95 (d, *J* = 8.0 Hz, 2H); 7.89 (d, *J* = 8.0 Hz, 2H); 7.80 (d, *J* = 8.0 Hz, 2H); 7.70 (s, 1H); 7.67 (s, 1H); 7.56 (t, *J* = 8.0 Hz, 2H); 7.45 (d, *J* = 8.0 Hz, 2H); 7.23 (t, *J* = 8.0 Hz; 1H); 7.16 (s, 1H); 4.74 (t, *J* = 6.0 Hz, 2H); 4.60 (t, *J* = 6.0 Hz, 2H); 3.78 (s, 3H). ^13^C NMR (DMSO-*d*_6_, 150.9 MHz) *δ* (ppm): 157.83, 155.81, 154.80, 154.51, 153.70, 152.89, 152.73, 152.09, 151.09, 147.46, 147.23, 143.03, 141.16, 136.73, 133.74, 132.49, 129.98, 129.23, 128.23, 126.38, 125.91, 123.63, 122.54, 121.47, 121.42, 121.34, 118.86 118.74, 116.63, 116.49, 108.57, 107.88, 104.47, 68.09, 56.08, 48.07, ESI-MS (*m/z*): found 498.183, (M^2+^, C_41_H_31_BrClFN_8_O_2_Pt requires 498.053); 1141.368, ([M+PF_6_]^+^, C_41_H_31_BrClF_7_N_8_O_2_PPt requires 1141.070).

Complex **4**. 138 mg, 71%. Elem. anal. calcd. (%) for C_42_H_33_BrClF_13_N_8_O_2_P_2_Pt·3H_2_O: C, 37.22; H, 2.90; N, 8.27; found: C, 37.29; H, 2.77; N, 8.48. ^1^H NMR (CD_3_CN, 400 MHz) *δ*(ppm): 8.54 (s, 1H); 8.45 (s, 2H); 8.39 (s, 1H); 8.33 (d, *J* = 8.0 Hz, 2H); 8.27 (t, *J* = 8.0 Hz, 2H); 8.14 (s, 1H); 8.06 (s, 1H); 7.93 (d, *J* = 8.0 Hz, 2H); 7.88 (d, *J* = 8.0 Hz, 2H); 7.77 (d, *J* = 8.0 Hz, 2H); 7.64 (s, 2H); 7.54 (t, *J* = 8.0 Hz, 2H); 7.41 (d, *J* = 8.0 Hz, 2H); 7.08 (s, 2H); 4.55 (t, *J* = 8.0 Hz, 2H); 4.19 (t, *J* = 8.0 Hz, 2H); 3.90 (s, 3H); 2.50 (t, *J* = 8.0 Hz, 2H). ^13^C NMR (DMSO-*d*_6_, 150.9 MHz) *δ* (ppm): 157.74, 155.79, 154.80, 153.75, 152.81, 152.72, 152.14, 151.28, 147.92, 147.20, 142.90, 140.52, 136.69, 133.74, 132.47, 129.96, 129.20, 128.47, 126.28, 125.87, 123.10, 122.69, 121.59, 121.55, 121.32, 118.87, 118.74, 116.58, 116.45, 108.59, 107.73, 104.53, 66.80, 55.97, 45.60, 28.90. ESI-MS (*m/z*): found 505.151, (M^2+^, C_42_H_33_BrClFN_8_O_2_Pt requires 505.061); 1155.299, ([M+PF_6_]^+^, C_42_H_33_BrClF_7_N_8_O_2_PPt requires 1155.086).

**References:**

Du, J., Kang, Y., Zhao, Y., Zheng, W., Zhang, Y., Lin, Y.*, et al.* (2016). Synthesis, Characterization, and in Vitro Antitumor Activity of Ruthenium(II) Polypyridyl Complexes Tethering EGFR-Inhibiting 4-Anilinoquinazolines. Inorg Chem *55*, 4595-4605.

Ji, L., Zheng, W., Lin, Y., Wang, X., Lü, S., Hao, X.*, et al.* (2014). Novel ruthenium complexes ligated with 4-anilinoquinazoline derivatives: Synthesis, characterisation and preliminary evaluation of biological activity. Eur J Med Chem *77*, 110-120.
